# Factors associated with hematoma expansion in deep versus lobar intracerebral haemorrhage: a multicentre observational study

**DOI:** 10.1093/esj/aakag002

**Published:** 2026-02-17

**Authors:** Francesco Arba, Jawed Nawabi, Qi Li, Andrea Dell’Orco, Giovanni Baronchelli, Federico Mazzacane, Giorgio Busto, Anna Cavallini, Francesco Palmerini, Maurizio Paciaroni, Michele Laudisi, Ilaria Casetta, Simona Sacco, Francesca Gabriele, Matteo Paolucci, Stefano Forlivesi, Mariarosaria Valente, Giovanni Merlino, Enrico Fainardi, Alessandro Padovani, Andrea Zini, Andrea Morotti

**Affiliations:** Stroke Unit, Careggi University Hospital, Florence, Italy; Department of Neuroradiology, Charité - Universitätsmedizin Berlin, Campus Mitte, Humboldt-Universität zu Berlin, Freie Universität Berlin, Berlin Institute of Health, Berlin, Germany; Department of Neurology, The Second Affiliated Hospital of Anhui Medical University, Hefei, China; Department of Neuroradiology, Charité - Universitätsmedizin Berlin, Campus Mitte, Humboldt-Universität zu Berlin, Freie Universität Berlin, Berlin Institute of Health, Berlin, Germany; Department of Clinical and Experimental Sciences, Neurology Unit, University of Brescia, Brescia, Italy; Department of Emergency Neurology and Stroke Unit, IRCCS Mondino Foundation, Pavia, Italy; Department of Biomedical Experimental and Clinical, Neuroradiology, University of Firenze, Careggi University Hospital, Florence, Italy; Department of Emergency Neurology and Stroke Unit, IRCCS Mondino Foundation, Pavia, Italy; Neurology Unit, Fondazione Poliambulanza, Istituto Ospedaliero, Brescia, Italy; Department of Neurosciences and Rehabilitation, Azienda Ospedaliero-Universitaria di Ferrara, Arcispedale Sant'Anna – Cona, University of Ferrara, Ferrara, Italy; Department of Neurosciences and Rehabilitation, Azienda Ospedaliero-Universitaria di Ferrara, Arcispedale Sant'Anna – Cona, University of Ferrara, Ferrara, Italy; IRCCS San Camillo Hospital, Venice, Italy; Department of Biotechnological and Applied Clinical Sciences, University of L'Aquila, L'Aquila, Italy; Department of Biotechnological and Applied Clinical Sciences, University of L'Aquila, L'Aquila, Italy; UOC Neurologia e Rete Stroke Metropolitana, IRCCS Istituto delle Scienze Neurologiche di Bologna, Ospedale Maggiore, Bologna, Italy; UOC Neurologia e Rete Stroke Metropolitana, IRCCS Istituto delle Scienze Neurologiche di Bologna, Ospedale Maggiore, Bologna, Italy; SOSD Stroke Unit, Department of Head, Neck, Neuroscience, Udine University Hospital, Udine, Italy; SOSD Stroke Unit, Department of Head, Neck, Neuroscience, Udine University Hospital, Udine, Italy; Department of Biomedical Experimental and Clinical, Neuroradiology, University of Firenze, Careggi University Hospital, Florence, Italy; Department of Clinical and Experimental Sciences, Neurology Unit, University of Brescia, Brescia, Italy; UOC Neurologia e Rete Stroke Metropolitana, IRCCS Istituto delle Scienze Neurologiche di Bologna, Ospedale Maggiore, Bologna, Italy; Department of Clinical and Experimental Sciences, Neurology Unit, University of Brescia, Brescia, Italy

**Keywords:** intracerebral haemorrhage, haematoma expansion, haemorrhage location, computed tomography

## Abstract

**Introduction:**

Identification of factors associated with haematoma expansion (HE) in patients with primary intracerebral haemorrhage (ICH) is crucial for optimization of management and therapeutic strategies. We investigated whether such factors differed according to supratentorial ICH location, comparing deep versus lobar ICH.

**Methods:**

Retrospective analysis of patients with primary ICH admitted at nine sites. HE was defined as growth ≥6 mL and/or ≥33% from baseline to follow-up imaging. We evaluated independent associations using multivariable logistic regression models adjusted for age, sex, baseline haematoma volume, anticoagulants and antiplatelets use and other relevant confounders identified in univariate analyses.

**Results:**

A total of 1768 patients were included (mean age 70 years, 56% males) of whom 1020 (58%) had deep and 748 (42%) had lobar ICH; HE occurred in 531 (30%) patients (28% deep and 33% lobar ICH). Age and baseline haematoma volume were shared predictors of HE in lobar and deep ICH. Anticoagulant use (OR = 1.61;95%, 1.04–2.50) and lower Glasgow Come Scale (OR = 0.91;95%CI, 0.85–0.96) were associated with HE only in lobar ICH, whereas the associations between systolic blood pressure >140 mmHg (OR = 1.53;95%CI, 1.03–2.29) and presentation before 3 h from onset (OR = 1.40;95%CI, 1.02–1.92) and HE were observed only in patients with deep ICH.

**Conclusions:**

Some factors associated with HE were shared between deep and lobar ICH whereas others appeared to be location-specific. Our findings may reflect differences in the pathophysiology of HE according to ICH location and might improve the stratification of HE risk in clinical practice or randomized trials.

## Graphical Abstract

**Graphical Abstract:**
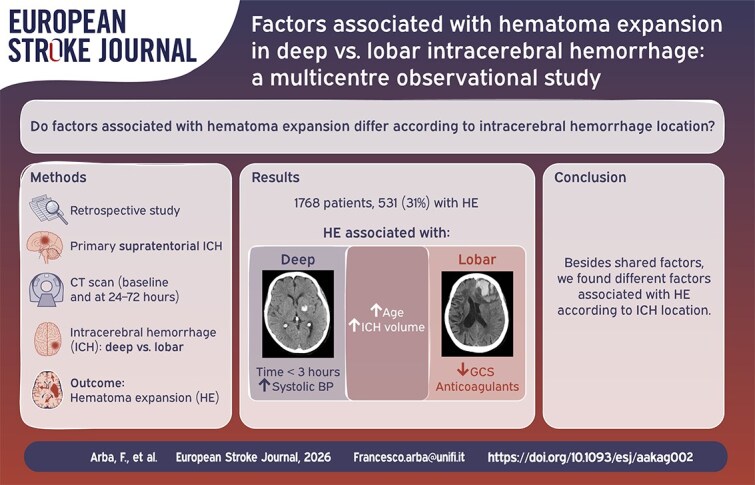

## Introduction

Intracerebral haemorrhage (ICH) is a devastating disease associated with high mortality and disability and represents a major challenge for health systems worldwide.^1^ Haematoma expansion (HE) is a common event in the early natural history of the disease and represents a major determinant of poor functional outcome and has been identified as one of the main therapeutic targets.^2^ Observational studies and pooled analyses suggested that some variables, such as baseline haematoma volume, time from symptom onset to computed tomography (CT) and antithrombotic treatment are independent predictors of HE.^3^

However, the mechanisms underlying HE have not been fully elucidated, particularly, it remains unclear whether predictors of HE differ according to ICH location and the underlying small vessel disease pathology. Deep ICH is typically associated with arteriolosclerosis and traditional vascular risk factors such as hypertension and diabetes. Conversely, cerebral amyloid angiopathy is the main cause of ICH occurring in cortical–subcortical (ie, lobar) location.^4^

As outlined by the American Heart Association ICH Guidelines,^5^ there is a need of a deeper understanding of HE pathophysiology in order to develop targeted therapeutic strategies. However, there is scarce information on whether factors associated with HE are shared between deep and lobar ICH or whether they may differ and be influenced by bleeding location. We tested the hypothesis that some predictors of HE might differ according to ICH location, comparing deep versus lobar supratentorial ICH.

## Methods

This study adheres to the STROBE (Strengthening the Reporting of Observational studies in Epidemiology) guidelines for observational studies (Supplementary Material). Patients were retrospectively selected from the pool of consecutive subjects admitted for acute ICH at the following academic medical institutions in a fifteen years period (2010–2024): Charité Hospital, Berlin, Germany; Spedali Civili, Brescia, Italy; Fondazione Poliambulanza, Brescia, Italy; Arcispedale S. Anna, Ferrara, Italy; IRCCS Mondino Foundation, Pavia, Italy; IRCCS Istituto delle Scienze Neurologiche, Bologna, Italy; San Salvatore Hospital, L’Aquila, Italy; Santa Maria della Misericordia Hospital, Udine, Italy and The First Affiliated Hospital of Chongqing Medical University, Chongqing, China. The Institutional review boards and ethical committees of each participating centre approved all the study procedures. Written informed consent was obtained by patients or family members or waived by the institutional review board according to each centre’s practice.

We applied the following inclusion criteria:(1) diagnosis of primary, spontaneous supratentorial ICH; (2) baseline non-contrast computed tomography (NCCT) obtained within 24 h from known onset time; (3) availability of follow-up NCCT; (4) age *>* 18. We excluded subjects with any of the following: (1) ICH secondary to vascular malformation, brain tumour or other intracranial disease detectable with angio-CT/angioMR, according to local centre’s protocol; (2) surgery before follow-up imaging; (3) infratentorial ICH; (4) patients with missing location/missing follow-up imaging. Clinical management followed the American Heart Association/American Stroke Association (AHA/ASA) guidelines and European Stroke Organisation (ESO) guidelines.^5-7^

### Clinical variables

Clinical variables of interest were age, sex, history of cardiovascular risk factors, antiplatelet and antithrombotic treatment, admission systolic and diastolic blood pressure, Glasgow Coma Scale (GCS), time from stroke onset to NCCT. All variables were collected by trained investigators through retrospective review of all available medical reports and were blinded to the outcomes of interest.

### Imaging variables

NCCT images were acquired at each participating site according to the local protocols. Baseline NCCT was performed within 24 h form symptoms onset or time last seen well, and follow-up NCCT was repeated at 24–72 h or earlier in case of clinical deterioration. ICH volume was measured with semi-automated volumetric softwares (ITK, https://itk.org/Horos, https://horosproject.org). ICH location was classified as supratentorial lobar (involving the cortex or subcortical white matter) and supratentorial deep (involving the deep nuclei, thalamus and deep white matter).^1^ HE was our outcome of interest and was defined as haematoma growth *>* 6 mL and/or *>*33%.^8^ The inter-rater reliability was tested in a subgroup of patients between two Neuroradiologists (EF, GB) and two Neurologists (AM, FM) with good agreement (Cohen’s K and IC > 0.80).

### Statistical analysis

Categorical variables were expressed as count (%) and compared with χ^2^ test. Continuous variables were expressed as mean (Standard Deviation) median (interquartile range), as appropriate, and compared with Student’s *t*-test and Mann–Whitney test, respectively. If baseline or follow-up missing data were present in more than 10% of the population we performed comparison between patients with and without data to detect attrition bias. To investigate independent associations, we built logistic regression models with HE as dependent variable adjusting for age, sex, baseline haematoma volume, time from symptoms onset to CT, antiplatelets, anticoagulants and for all variables with *P* <.1 at univariate analysis. Statistical significance was set at *P* <.05 and all the analyses were performed using SPSS for Mac (version 25.0; SPSS, IBM Corp., Armonk, NY).

## Results

### General characteristics

**Figure 1:**
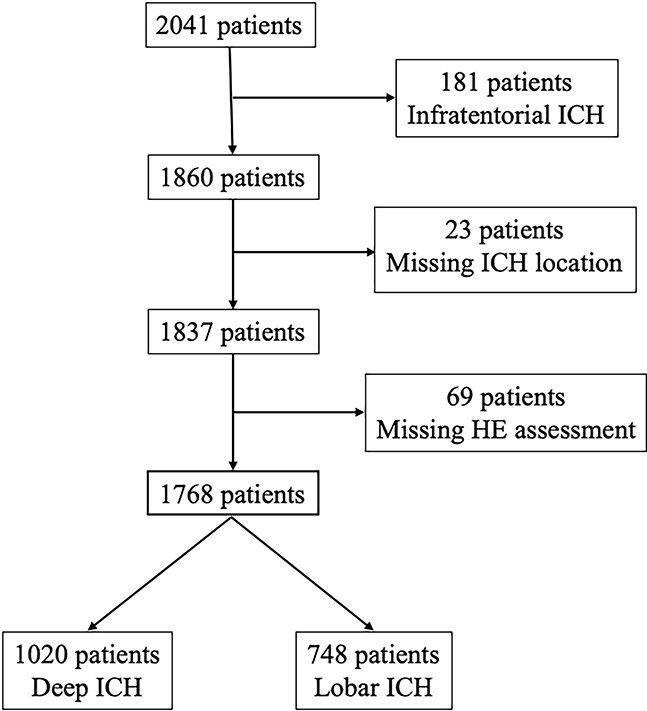
Flow chart of study population.

We identified a total of 2041 patients, of whom 1768 met the inclusion criteria. The study selection flowchart is illustrated in Figure 1. Among included subjects, 1020 (58%) had a deep and 748 (42%) and a lobar ICH location.

The general characteristics of study population are shown in Table 1. Compared to patients with lobar ICH, those with deep ICH were younger (68.7 vs 72.6 years, *P* <.001), more frequently male (60% vs 50%, *P* <.001), had higher systolic (167 vs 161 mmHg, *P* <.001) and diastolic (93 mmHg vs 88 mmHg, *P* <.001) blood pressure, lower baseline ICH volume (11.5 mL vs 25.5 mL, *P* <.001), and had higher prevalence of hypertension.

**Table 1:** General characteristics of study population stratified by ICH location.

**Variables**	**Total** ***n* = 1768**	**Deep** ***n* = 1020**	**Lobar** ***n* = 748**	***P***
**Age, years, mean (±SD)**	70.4 (±13.5)	68.7 (±13.8)	72.6 (±12.6)	**<.001**
**Sex, male**	990 (56)	614 (60)	376 (50)	**<.001**
**Systolic BP, mHg, mean (±SD)**	164.7 (±31.0)	167.2 (±30.8)	161.3 (±30.9)	**<.001**
**Diastolic BP, mmHg, mean (±SD)**	90.7 (±17.6)	92.6 (±17.7)	88.0 (±16.9)	**<.001**
**GCS, median (IQR)**	14 (11–15)	14 (11–15)	14 (11–15)	.271
**Time to NCCT, median (IQR) min** ^a^	240 (128–656)	213 (120–600)	300 (144–720)	**<.001**
**Time <3 h** ^a^	666 (40)	431 (45)	235 (34)	**<.001**
**Hypertension**	1293 (73)	766 (75)	527 (70)	**.030**
**Diabetes**	342 (19)	208 (20)	134 (18)	**.193**
**Antiplatelets**	467 (26)	247 (24)	220 (29)	**.014**
**Anticoagulants**	342 (19)	183 (18)	159 (21)	.081
**ICH volume median (IQR), median, mL**	15.3 (6.6–36.2)	11.5 (5.5–25.6)	25.5 (10.3–49.5)	**<.001**
**Absolute haematoma growth, median, mL**	7.58 (±31.5)	4.87 (±14.8)	9.05 (±46.03)	**<.001**
**Intraventricular haemorrhage**	580 (35)	379 (37)	201 (27)	**<.001**
**HE**	531 (30)	283 (28)	248 (33)	**.014**

Abbreviations: BP = blood pressure; GCS = Glasgow Coma Scale; HE = haematoma expansion; ICH = intracerebral haemorrhage; IQR = interquartile range; NCCT = non-contrast computed tomography; SD = standard deviation.

^a^114 patient’s data missing. Data with *p* < 0.05, i.e. statistical significance.

### HE in lobar and deep ICH

Overall, 531 (30%) patients had HE, 283 (23%) in deep and 248 (33%) in lobar ICH (*P* =.014). In deep ICH, patients with HE were older (71.4 vs 67.2 years, *P* <.001), had more frequently hypertension (79 % vs 74%, *P* =.027) and were more frequently under antiplatelet treatment (29% vs 23%, *P* =.042) (Table 2). Deep ICH subjects with HE had higher systolic blood pressure (170 mmHg vs 164 mmHg, *P* =.007) and had more frequently onset to CT time within 3 h (50% vs 42%, *P* =.027). In lobar ICH, patients with HE were older (74.4 vs 70.6, *P* <.001), more frequently under anticoagulant treatment (27% vs 17%, *P* =.012). In patients with HE, baseline haematoma volume was higher, GCS and time from symptoms onset to NCCT were lower in patients with both deep and lobar ICH (Tables 2 and 3).

**Table 2:** General characteristics of patients with deep intracerebral haemorrhage with and without haematoma expansion.

**Variables**	**Total** ***N* = 1020**	**Haematoma expansion − *n* = 737**	**Haematoma expansion + *n* = 283**	***P***
**Age, years, mean (±SD)**	68.7 (±13.8)	67.4 (±14.5)	71.3 (±11.6)	**<.001**
**Sex, male**	614 (60)	445 (60)	169 (60)	.847
**Systolic BP, mean (±SD), mmHg**	161.3 (±30.9)	164.4 (±31.2)	170.9 (±29.8)	**.005**
**Systolic BP > 140 mmHg**	804 (79)	568 (77)	236 (83)	**.029**
**Diastolic BP, mean (±SD), mmHg**	92.6 (±17.8)	92.1 (±17.9)	92.2 (±15.7)	.913
**GCS, median (IQR)**	14 (11–15)	14 (11–15)	13 (9–15)	**<.001**
**Time to NCCT, median (IQR), minutes**	213 (120–600)	243 (126–707)	195 (117–420)	**<.001**
**Time < 3 h**	431 (45)	296 (42)	135 (50)	**.027**
**Hypertension**	766 (75)	542 (74)	224 (79)	**.064**
**Diabetes**	208 (20)	151 (21)	57 (20)	.902
**Antiplatelets**	247 (24)	166 (23)	81 (29)	**.042**
**Anticoagulants**	183 (18)	123 (17)	60 (21)	**.093**
**ICH volume, median (IQR), mL**	11.5 (5.5–25.6)	10.4 (5.3–21.9)	17.6 (6.6–44.9)	**<.001**

Abbreviations: BP = blood pressure; GCS = Glasgow Coma Scale; HE = haematoma expansion; ICH=intracerebral haemorrhage; IQR = interquartile range; NCCT = non-contrast computed tomography; SD = standard deviation. Data with *p* < 0.05, i.e. statistical significance.

**Table 3:** General characteristics of patients with lobar intracerebral haemorrhage with and without haematoma expansion.

**Variables**	**Total** ***n* = 748**	**Haematoma Expansion—*n* = 500**	**Haematoma Expansion +** ***n* = 248**	***P***
**Age, years, mean (±SD)**	72.4 (±12.7)	70.6 (±13.6)	74.5 (±10.9)	**<.001**
**Sex, male**	376 (50)	245 (49)	131 (53)	.420
**Systolic BP, mean (±SD), mmHg,**	161.4 (±30.9)	160.3 (±31.2)	162.8 (±30.2)	.306
**Systolic BP > 140 mmHg**	533 (71)	344 (69)	189 (76)	**.035**
**Diastolic BP, mean (±SD), mmHg**	88.1 (±16.9)	87.7 (±17.1)	88.8 (±16.3)	.392
**GCS, median (IQR)**	14 (11–15)	14 (11–15)	12 (9–15)	**<.001**
**Time to NCCT, median (IQR), minutes**	300 (144–720)	330 (155–840)	280 (150–592)	**.020**
**Time < 3 h**	235 (34)	146 (32)	89 (39)	**.057**
**Hypertension**	527 (71)	346 (69)	181 (73)	.286
**Diabetes**	134 (18)	88 (18)	46 (19)	.750
**Antiplatelets**	220 (29)	136 (27)	84 (34)	**.059**
**Anticoagulants**	159 (21)	93 (17)	66 (27)	**.012**
**ICH volume, median (IQR), ml**	25.5 (10.3–49.5)	18.7 (7.8–38.3)	40.4 (16.4–68.5)	**<.001**

Abbreviations: BP = blood pressure; GCS = Glasgow Coma Scale; HE = haematoma expansion; ICH=intracerebral haemorrhage; IQR = interquartile range; NCCT = non-contrast computed tomography; SD = standard deviation. Data with *p* < 0.05, i.e. statistical significance.

**Figure 2:**
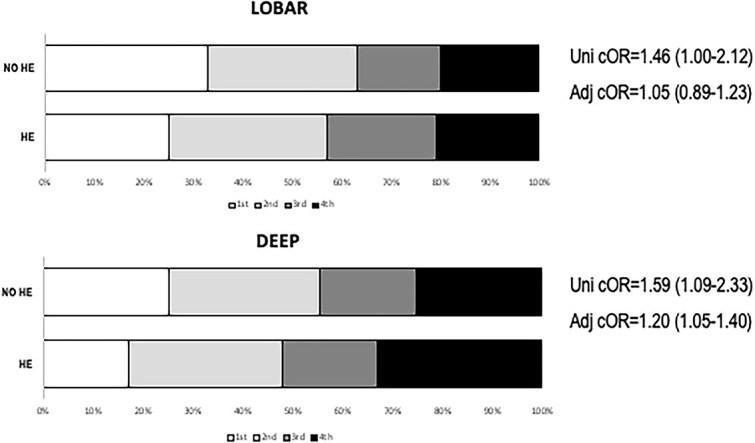
Association between systolic blood pressure quartiles and haematoma expansion stratified for haematoma location. Multivariate analyses are adjusted for age, sex, time from symptoms onset to CT, antiplatelets, anticoagulants.

In multivariable logistic regression models (Table 4), age and baseline haematoma volume were associated with HE in both lobar and deep ICH in all models. In lobar ICH, GCS (OR = 0.91; 95%CI, 0.85–0.96) and anticoagulants (OR = 1.61; 95%CI, 1.04–2.50) were associated with HE, whereas in deep ICH time from onset to CT <3 h and each 10-mmHg increase of systolic blood pressure were associated with an increase of the risk of HE of 40% (OR = 1.40; 95%CI, 1.02–1.92) and 8% (OR = 1.08; 95%CI, 1.02–1.14), respectively. Further analysis in deep ICH showed that systolic blood pressure > 140 mmHg was associated with HE (OR = 1.61; 95%CI, 1.08–2.42) and a dose–response effect of systolic blood pressure quartiles with HE (OR = 1.20 per-blood pressure quartile; 95%CI, 1.05–1.40; Figure 2).

**Table 4:** Multivariable logistic regression analysis in patients with lobar and deep intracerebral haemorrhage with haematoma expansion as dependent variable.

	**Lobar**	**Deep**
**Variables**	**OR (95% CI)**	**OR (95% CI)**
**Age**	**1.02 (1.00–1.04)**	**1.02 (1.01–1.04)**
**ICH volume, per ml increase**	**1.02 (1.01–1.02)**	**1.02 (1.01–1.03)**
**GCS, per-point decrease**	**1.09 (1.04–1.16)**	1.02 (0.97–1.06)
**Time < 3 h**	1.16 (0.79–1.71)	**1.40 (1.02–1.92)**
**Antiplatelets**	1.22 (0.82–1.82)	1.24 (0.87–1.76)
**Anticoagulants**	**1.61 (1.04–2.50)**	1.04 (0.68–1.59)
**SBP, per 10 mmHg increase**	-	**1.08 (1.02–1.14)**
**Systolic BP > 140 mmHg**	1.34 (0.90–2.01)	**1.53 (1.03–2.29)** ^a^
**Hypertension**	-	1.14 (0.78–1.67)

Abbreviations: BP=blood pressure; CI=confidence interval; GCS = Glasgow Coma Scale; ICH = intracerebral haemorrhage; OR = odd ratio. Analysis adjusted for age, sex, time from symptoms onset to CT, antiplatelets, anticoagulants and all variables listed in the table.

^a^Analysis adjusted for all variables listed in the table except SBP. Data with *p* < 0.05, i.e. statistical significance.

## Discussion

In patients with primary small vessel disease-related ICH, we found differences in factors associated with HE between lobar and deep ICH. Some predictors of HE such as age and baseline volume were shared; while others, such as stroke severity and anticoagulation in lobar ICH and systolic blood pressure and early presentation in deep ICH were location-specific.

Our findings raise the intriguing hypothesis of different biological mechanisms underlying HE according to ICH location and underlying cerebral small vessel disease.

Previous studies demonstrated differences in the frequency and timing of HE in deep versus lobar ICH.^9,10^ We found that shorter time from onset to CT time was not associated with HE in lobar rather with HE in deep ICH, in keeping with robust data from a large individual patient data meta-analysis.^3^ Similarly, previous observations highlighted a better diagnostic performance of imaging markers of HE within few hours from symptoms onset.^11,12^ This may suggest a different biology and timing of underlying HE in deep versus lobar ICH. This hypothesis is in line with a recent report that demonstrated that in lobar ICH due to cerebral amyloid angiopathy, HE might occur in a delayed fashion and over a longer time window.^13^ Stroke severity, expressed with GCS, was associated with HE in lobar but not in deep ICH. Previous studies showed an improvement in diagnostic accuracy for HE prediction when accounting for stroke severity, and a correlation between GCS and haematoma volume has been reported.^14,15^ Stroke severity might therefore simply be an epiphenomenon of ICH volume, an established predictor of HE.^3^ However, our findings do not support this hypothesis as GCS and haematoma volume were both independent predictors of HE in lobar ICH. The association between anticoagulation and HE only in lobar ICH might suggest a greater susceptibility to antithrombotic treatment and higher risk of bleeding in subjects with CAA, one of the main causes of lobar ICH.^16,17^ Although a recent study does not seem to confirm our observations,^18^ such discrepancy might be explained by heterogeneity in study cohorts, differences in type of anticoagulant medications and lack of magnetic resonance data in our cohort to confirm the underlying etiological subtype of small vessel disease.

Another interesting finding of our analysis is that systolic blood pressure higher than 140 mmHg was associated with HE only in deep ICH. We also observed a linear dose–response increase in the risk of deep HE across blood pressure quartiles, supporting a causal relation between higher systolic blood pressure and HE in deep ICH. In line with our results, a recent secondary analysis of the ATACH-II trial^19^ reported a different response to intensive systolic blood pressure (SBP) lowering in deep versus lobar ICH, with intensive SBP reduction associated with lower odds of HE only in deeply located ICH, reinforcing the concept that aetiology of ICH and pathophysiology of HE might differ according to ICH location. Different brain (deep vs cortical and leptomeningeal) circulation physiology and regulatory mechanisms may be at least in part responsible of such findings that need confirmation in future studies.

Our findings might have relevant implications for clinical practice and clinical trials. HE is a key therapeutic target and a better knowledge of the differences in HE between deep and lobar ICH might improve the selection of patients in the setting of randomized controlled trials. Location-specific differences in HE might also lead to a more accurate identification of ICH patients likely to derive benefit from specific medical therapies targeting HE.^19^

Our study has limitations. First, the retrospective design of our study might have introduced bias and does not allow causal associations. Selection bias could not be excluded, since patients with very severe ICH may lack follow-up imaging, as well as patients with very small haemorrhages and few neurological symptoms. Moreover, some patients may lack follow-up CT scan in favour of MRI to investigate haemorrhage aetiology. However, missing follow-up CT scans constitute a residual part of our clinical practice. Again, we could not account for more specific variables associated with HE, such as blood pressure fluctuations, time to blood pressure target, timing of coagulopathy reversal, haemoglobin levels,^20,21^ and other definitions of HE were not explored in our study.^22^ Thirdly, we did not systematically perform brain MRI or other biomarkers, thus we were unable to characterize the underlying small vessel disease and the relation with haemorrhage location. Again, the study population was enrolled in a long-time frame, during which standards of care changed. Therefore, we cannot rule out that different recommendations over time, such as systolic blood pressure targets and coagulopathy reversal, might have influenced our results. For the same reason, new emerging factors associated with HE, such as lower haemoglobin levels, have not been systematically recorded in our dataset, therefore we lack control on potentially meaningful factors associated with the outcome of interest. Among strengths of the study should be considered the multicentre design of our study, the relevant sample size, the low attrition of the cohort, and the standardized assessment of HE.

In conclusion, our results showed that there are shared factors associated with HE in lobar and deep ICH, such as age and baseline haematoma volume, but also different factors according to location, possibly reflecting substantial differences in pathophysiology of HE. Our findings support the hypothesis that mechanisms underlying HE may differ between lobar and deep ICH, suggesting that therapeutic strategies might be targeted according to ICH location. Further validation studies are warranted.

## Supplementary Material

aakag002_Supplemental_Material_STROBE_checklist_ICH

## Data Availability

The data supporting the findings of this study are available from the corresponding author upon reasonable request.
