# Supplementary material for: Factors associated with hematoma expansion in deep versus lobar intracerebral haemorrhage: a multicentre observational study
Source: Eur Stroke J. 2026 Feb 17;11(2):aakag002. doi: 10.1093/esj/aakag002 (PMC12911930; doi:10.1093/esj/aakag002)
Supplement: aakag002_Supplemental_Material_STROBE_checklist_ICH [file aakag002_supplemental_material_strobe_checklist_ich.doc]

STROBE Statement—checklist of items that should be included in reports of observational studies

|  | Item No | Recommendation |
| --- | --- | --- |
| **Title and abstract** | 1 | (*a*) Indicate the study’s design with a commonly used term in the title or the abstract (1) |
|  |  | (*b*) Provide in the abstract an informative and balanced summary of what was done and what was found (3) |
| Introduction | | |
| Background/rationale | 2 | Explain the scientific background and rationale for the investigation being reported (4) |
| Objectives | 3 | State specific objectives, including any prespecified hypotheses (4) |
| Methods | | |
| Study design | 4 | Present key elements of study design early in the paper (4-5) |
| Setting | 5 | Describe the setting, locations, and relevant dates, including periods of recruitment, exposure, follow-up, and data collection (5-6) |
| Participants | 6 | (*a*) *Cohort study*—Give the eligibility criteria, and the sources and methods of selection of participants. Describe methods of follow-up (5-6)  *Case-control study*—Give the eligibility criteria, and the sources and methods of case ascertainment and control selection. Give the rationale for the choice of cases and controls  *Cross-sectional study*—Give the eligibility criteria, and the sources and methods of selection of participants |
|  |  | (*b*) *Cohort study*—For matched studies, give matching criteria and number of exposed and unexposed (NA)  *Case-control study*—For matched studies, give matching criteria and the number of controls per case |
| Variables | 7 | Clearly define all outcomes, exposures, predictors, potential confounders, and effect modifiers. Give diagnostic criteria, if applicable (5-6) |
| Data sources/ measurement | 8* | For each variable of interest, give sources of data and details of methods of assessment (measurement). Describe comparability of assessment methods if there is more than one group (6) |
| Bias | 9 | Describe any efforts to address potential sources of bias ( |
| Study size | 10 | Explain how the study size was arrived at (NA) |
| Quantitative variables | 11 | Explain how quantitative variables were handled in the analyses. If applicable, describe which groupings were chosen and why (6) |
| Statistical methods | 12 | (*a*) Describe all statistical methods, including those used to control for confounding (6) |
|  |  | (*b*) Describe any methods used to examine subgroups and interactions (6) |
|  |  | (*c*) Explain how missing data were addressed (6) |
|  |  | (*d*) *Cohort study*—If applicable, explain how loss to follow-up was addressed (6)  *Case-control study*—If applicable, explain how matching of cases and controls was addressed  *Cross-sectional study*—If applicable, describe analytical methods taking account of sampling strategy |
|  |  | (*e*) Describe any sensitivity analyses (NA) |

Continued on next page

| Results | | |
| --- | --- | --- |
| Participants | 13* | (a) Report numbers of individuals at each stage of study—eg numbers potentially eligible, examined for eligibility, confirmed eligible, included in the study, completing follow-up, and analysed (7) |
|  |  | (b) Give reasons for non-participation at each stage (7) |
|  |  | (c) Consider use of a flow diagram (7, Figure 1) |
| Descriptive data | 14* | (a) Give characteristics of study participants (eg demographic, clinical, social) and information on exposures and potential confounders (7, Table 1) |
|  |  | (b) Indicate number of participants with missing data for each variable of interest (NA) |
|  |  | (c) *Cohort study*—Summarise follow-up time (eg, average and total amount) (NA) |
| Outcome data | 15* | *Cohort study*—Report numbers of outcome events or summary measures over time (7) |
|  |  | *Case-control study—*Report numbers in each exposure category, or summary measures of exposure |
|  |  | *Cross-sectional study—*Report numbers of outcome events or summary measures |
| Main results | 16 | (*a*) Give unadjusted estimates and, if applicable, confounder-adjusted estimates and their precision (eg, 95% confidence interval). Make clear which confounders were adjusted for and why they were included (7-8) |
|  |  | (*b*) Report category boundaries when continuous variables were categorized (NA) |
|  |  | (*c*) If relevant, consider translating estimates of relative risk into absolute risk for a meaningful time period (NA) |
| Other analyses | 17 | Report other analyses done—eg analyses of subgroups and interactions, and sensitivity analyses (NA) |
| Discussion | | |
| Key results | 18 | Summarise key results with reference to study objectives (8) |
| Limitations | 19 | Discuss limitations of the study, taking into account sources of potential bias or imprecision. Discuss both direction and magnitude of any potential bias (9-10) |
| Interpretation | 20 | Give a cautious overall interpretation of results considering objectives, limitations, multiplicity of analyses, results from similar studies, and other relevant evidence (9-10) |
| Generalisability | 21 | Discuss the generalisability (external validity) of the study results (9-10) |
| Other information | | |
| Funding | 22 | Give the source of funding and the role of the funders for the present study and, if applicable, for the original study on which the present article is based (10-11) |

*Give information separately for cases and controls in case-control studies and, if applicable, for exposed and unexposed groups in cohort and cross-sectional studies.
